# Mind the gap: bridging ethical considerations and regulatory oversight in implantable BCI human subjects research

**DOI:** 10.3389/fnhum.2025.1633627

**Published:** 2025-07-23

**Authors:** R. Bert Wilkins, Tara Coffin, Michelle Pham, Eran Klein, Megh Marathe

**Affiliations:** ^1^WCG Clinical, Cary, NC, United States; ^2^Center for Bioethics and Social Justice, Department of Medicine, Michigan State University, East Lansing, MI, United States; ^3^Department of Philosophy, University of Washington, Seattle, WA, United States; ^4^Department of Neurology, Oregon Health and Science University, Portland, OR, United States; ^5^Department of Media and Information, Center for Bioethics and Social Justice, Michigan State University, East Lansing, MI, United States

**Keywords:** neural implants, human subjects research, institutional review board (IRB), implantable BCI, ethical review, investigational device exemption (IDE), informed consent, legally authorized representatives

## Abstract

The advent of Brain-Computer Interface (BCI) technology brings groundbreaking advancements in medical science but also raises important ethical considerations. This manuscript explores the ethical dimensions of implantable BCIs (iBCIs), focusing on the central role of Institutional Review Boards (IRBs) in the United States, in safeguarding participant rights and welfare. As federally mandated bodies, IRBs ensure that informed consent is obtained ethically, emphasizing participant autonomy, preventing undue coercion, while supporting clear and practical communication of risks and benefits. As part of this discussion, this paper touches on the ethical challenges surrounding the enrollment of participants with impaired consent capacity and the long-term implications of implanted brain devices. Additionally, this work underscores the critical importance of robust cybersecurity measures to prevent data breaches and unauthorized manipulation of brain activity. By examining risk assessments, data management practices, and the need for external cybersecurity expertise, this work offers a comprehensive framework for IRB review of iBCI research. This perspective aims to guide ethical iBCI research and protect human subjects in this rapidly evolving field.

## Introduction

Brain-Computer Interfaces (BCIs) are advanced systems that facilitate direct communication between the brain and external devices, bypassing traditional neuromuscular pathways (Chaudhary et al., 2016). These systems can be both wearable and implanted. Implantable Brain-Computer Interfaces (iBCIs) are BCIs that are surgically placed into the brain, with the goal of enabling communication between the brain and an external device. Such interfaces offer transformative potential, from restoring or substituting for sensory and motor functions in patients with disabilities, to enhancing cognitive capabilities and revolutionizing human-technology interactions. Technological advances in the area of iBCIs have led to an increased interest in applying this innovative technology, highlighting critical challenges and regulatory considerations in the process (Rapeaux and Constandinou, 2021).

iBCIs can potentially address an unmet need in supporting daily living activities for individuals diagnosed with certain medical conditions, including stroke, paralysis, amyotrophic lateral sclerosis (ALS), traumatic brain injury, Parkinson’s disease, and multiple sclerosis (MS). These conditions can leave an individual with challenges around communication, mobility, as well as psychiatric and emotional wellbeing. Traditional assistive communication devices, such as keyboards or picture boards, or alternative means of communication, such as gestures or eye blinking, can be ineffective or onerous when motor disabilities accompany communication impairment. While newer assistive devices, like eye tracking software, can significantly enhance communication abilities, they can be difficult to learn to use and may compromise an individual’s autonomy and privacy (Baxter et al., 2012). By enabling independent communication, or controlling external devices including robotic arms, iBCIs have the potential to significantly enhance the quality of life for some individuals affected by various neurological conditions. Despite their broad applications, most iBCIs are considered investigational. Consequently, research in this burgeoning field presents distinct challenges for Institutional Review Boards (IRBs) responsible for regulatory review and oversight.

This article examines the ethical considerations the IRB should address when reviewing clinical research involving iBCIs. The iBCI-related ethical dilemmas that arise in the context of IRB review are underexplored. After surveying some of these ethical dilemmas, we will review the existing U.S. regulatory frameworks and guidelines designed to protect human participants in iBCI studies. Finally, we will outline some key criteria that oversight bodies, such as IRBs, should consider when evaluating research proposals involving iBCIs.

### Current U.S. regulations

**Figure 1:**
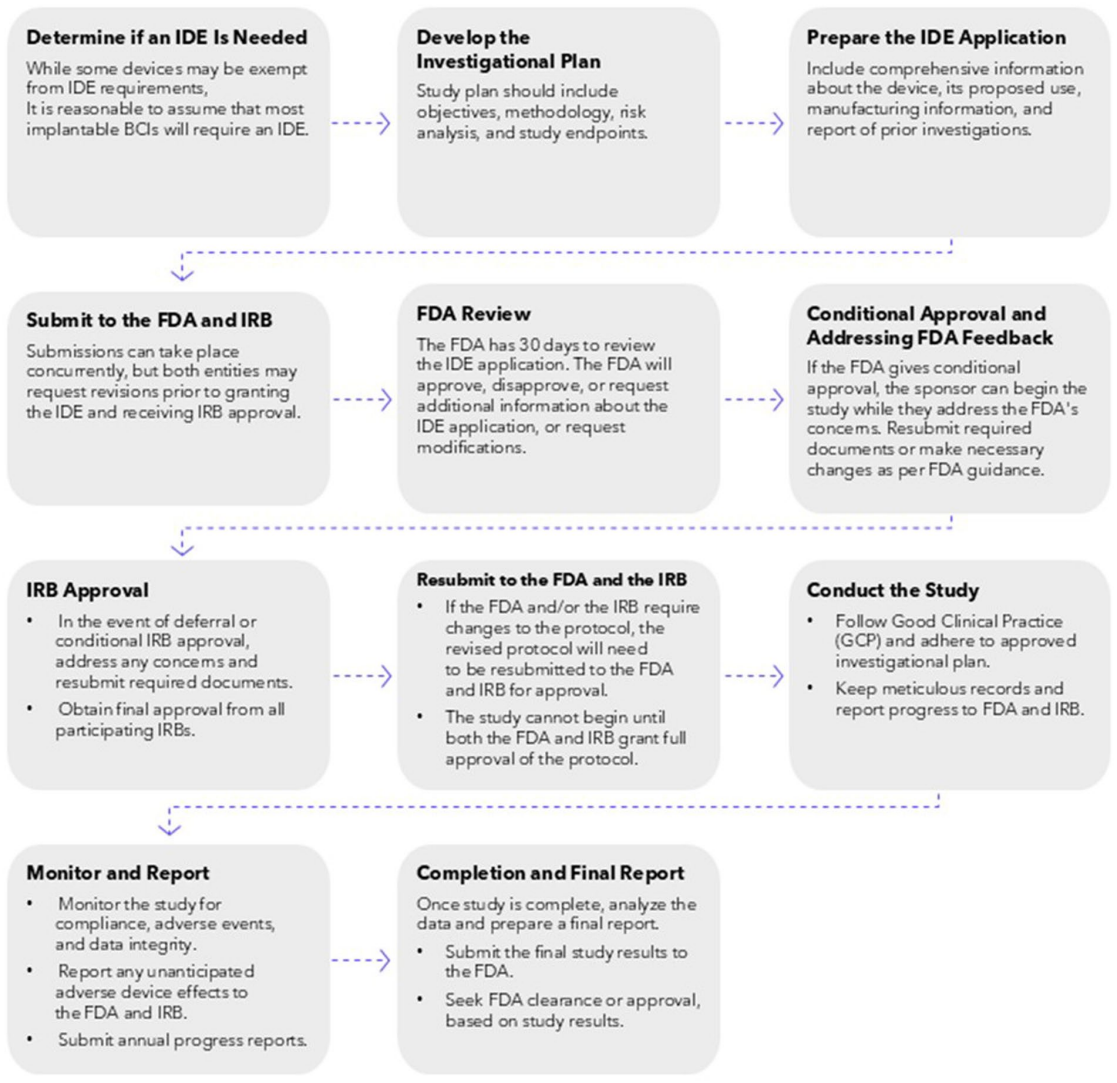
Required steps involved with requesting an IDE, conducting research that has received IRB approval, and submitting completed research analysis and data for FDA approval of an investigational iBCI device.

The U.S. Food and Drug Administration (FDA) regulates investigational medical devices under the Investigational Device Exemption (IDE) program (21 CFR 812). The IDE process involves a review of the device’s safety and efficacy, as well as a thorough examination of its design, materials, and clinical study protocols. The FDA reviews and approves the IDE application before clinical trials can begin, ensuring that any risks associated with device use are minimized for testing and that the study design is scientifically valid. Along with this initial oversight, any changes to the protocol must also be submitted to the FDA and the FDA may inspect, or audit, the study to ensure compliance with regulations (Figure 1).

In 2021, the FDA published formal guidance for iBCI devices, specifically for patients with paralysis or amputation (U.S. Food and Drug Administration, 2019). The guidance emphasizes the importance of providing clear and comprehensive information about the device, including its design, components, and function. It also highlights the need for thorough risk management and cybersecurity assessments. The document provides specific recommendations for non-clinical testing, including bench testing and animal studies, to evaluate the safety and efficacy of the device. It also outlines considerations for clinical performance testing, including patient selection, study design, and endpoints. Importantly, the guidance also speaks to the importance of human factors engineering to ensure the device is safe and user-friendly, with consideration for both the hardware and software components involved with iBCIs.

Once the sponsor has completed the clinical trials and has sufficient data to show efficacy and safety, they will apply for a Premarket Approval (PMA) from the FDA. The PMA, one route by which a device may receive approval for market use, is considered the most comprehensive medical device marketing submission (U.S. Food and Drug Administration, 2023a). With the PMA submission process, which is considered appropriate for high risk devices, review relies on the independent demonstration of safety and effectiveness of the device (U.S. Food and Drug Administration, 2023a). High risk medical devices, categorized as class III medical devices, include those that supports life, prevents impairment, or presents a significant risk of illness or injury to the user. Given the risks of an iBCI, including the surgery to implant the device, the risks or cyber attacks, and the possibility of long-term personality changes or neuronal activity, they are likely to always be considered a class III medical device and prior to being marketed, they will need to go through the IDE/PMA processes.

Current regulatory mechanisms tend to focus on premarket safety and efficacy, with less emphasis on long-term surveillance and post-market follow-up (Caughron and Dhruva, 2023). This is problematic for iBCIs, which may induce neural changes that unfold over extended periods, requiring monitoring protocols that are more persistent. As a result, there is a growing recognition that iBCIs may require a more specialized and adaptive regulatory approach—one that can address the multifaceted challenges and uncertainties surrounding these devices—than is currently in place.

### Role of the IRB

Clinical trials of products that fall under FDA oversight and involve human subjects must be reviewed by an IRB. The purpose of this review is to provide an independent appraisal of the research to determine if the study is compliant with federal regulations and that the rights and welfare of the participants are protected. While IRBs in the US are subject to the same set of regulations, individual IRBs may differ in areas of specialty, turnaround times, or scope of review. For example, IRBs based out of academic institutions focus on research conducted within their institutions, while commercial IRBs review studies conducted across multiple institutions and may have access to specialized staff and resources to specific research types.

As part of the independent review process, the IRB is required to be comprised of individuals from diverse backgrounds and perspectives. IRB members must include physicians, scientists, and non-scientists. Importantly, the IRB must also have sufficient expertise to evaluate the research either directly or in consultation with an expert. For iBCI studies, this would likely include a neurologist and/or a neurosurgeon.

When reviewing a research protocol, the IRB considers several issues related to the research. The first is a regulatory requirement. That is, does the study comply with the federal regulations? For example, for a study involving an investigational device, the IRB will determine if the appropriate device findings are in place, which may include an Investigational Device Exemption (IDE) from the FDA. Additionally, the IRB will also determine if the protocol adequately and accurately describes the research, and if the consent form contains all the required elements to promote informed consent.

The second role of the IRB is to determine if the risk–benefit ratio is acceptable. Generally, this means that the potential clinical and non-clinical benefits of the research outweigh the potential risks. This does not mean there cannot be any risks, but it does mean that the riskier the study, the more potential benefit there needs to be. There may be direct benefits to the participants due to increased mobility or improved communication, even if this is only within the context of the clinical study. This potential for direct benefit arguably justifies the risks associated with the use of iBCIs. Some studies cannot promise the possibility of direct benefit, such as feasibility or proof of principle studies, but provide potential generalizable benefits to society by getting more information on how iBCIs operate, their risks, and ultimately their approval.

If the IRB determines that the study is not acceptable, they can disapprove the study or require modifications to secure approval. The IRB will also have oversight of the study and review the research at least annually, for the entire duration of the study. As part of this oversight, the IRB reviews any changes to the protocol, consent form, or any other participant-facing documents, and continues to evaluate the risk profile of the study and any changes that could impact the rights and welfare of the participants.

Clinical trials of iBCIs pose challenges for IRBs that other clinical trials do not. The first is that while the number of iBCIs are increasing, the number of clinical trials, compared to other diseases or therapeutic areas, is still low. As such, the IRB does not have the opportunity to gain experience with these types of devices and are not able to see similar issues or study designs repeatedly. Without this invaluable experience, the IRB often must relearn what iBCIs are, and the unique ethical issues presented with each new study.

While the IRB is required to have access to the expertise necessary to conduct appropriate review, either through membership or via a consultation, the specific expertise necessary to review research involving iBCIs is difficult to come by. There is not a large pool of neurologists and/or neurosurgeons for IRBs to call upon who have experience or expertise with neural implants generally or iBCIs specifically. Having appropriate expertise is paramount in determining if the risks of the iBCI are outweighed by the benefits of the study. While some of the risks are more apparent, such as the surgical risks, others, like changes in personality or functionality are harder to define or are less well understood, making the evaluation of the risk–benefit ratio of the study challenging (Kubu et al., 2019; Okun et al., 2024).

In addition to the clinical aspects, the IRB also considers cybersecurity issues and determines if there are sufficient protections for the participants. Finding members or consultants with the appropriate expertise in cybersecurity can be time-consuming and can delay the start of the research. In addition to securing appropriate expertise, one of the central ways an IRB serves to protect participants, is to ensure that prospective participants have sufficient knowledge to determine if they want to be in the research, and that the consent process is appropriate, and that the autonomy of the individual is respected.

### Informed consent for iBCI research

One of the primary ethical dimensions of conducting human subjects research on iBCIs is the nuances around informed consent. It is challenging to communicate benefits and risks associated with iBCIs, including uncertainties about long-term effects, threats to personhood, and concerns around privacy and data security (Ploesser et al., 2024). In this section, we will discuss these ethical considerations, highlighting challenges and proposing strategies to address them within the context of clinical research, while focusing on the role of IRBs to preserve patient safety and welfare. IRB oversight is intended to complement FDA guidance and regulations, reviewing investigative device studies to ensure that they meet the regulatory requirements and ethical standards for human subjects research. This includes supporting informed consent.

The objective of informed consent is to ensure that research participation is voluntary and based on a full understanding of the nature of the research and the participant’s role in it (21 CFR 50.25). This process can be challenging in the context of iBCI research, as the complexity of emerging technology can be difficult to communicate, public knowledge about neurotechnology is limited, and risks can be difficult to conceptualize. In addition, prospective participants may have expectations for therapeutic benefit even in proof-of-concept or early feasibility studies (Klein, 2016). A participant may experience functional improvements during the study (e.g., faster communication, improved prosthetic functionality) that are not sustainable or available outside of the research setting.

Even if the consent process accurately portrays the abilities and the limitations of the iBCI, participants may harbor an overly optimistic view of the iBCI, or generally lack of practical understanding of the technology, raising doubts about the validity of the informed consent. For example, an individual who is desperate for relief or improvement in their condition may ignore or inappropriately discount study information about risk. Alternatively, the voluntariness of informed consent could come into question if participants feel pressured to participate in a study in hopes that functional improvement would lessen the burden on a caregiver.

Risk information is difficult to appropriately communicate. Beyond the routine risks of undergoing surgery or taking part in a research study, iBCIs pose a unique risk to an individual’s sense of self and autonomy (Table 1). Current research indicates that some iBCIs have the potential to alter an individual’s cognitive and psychological state, which may erode their sense of self and personal autonomy (Kenneally, 2021). For example, Sample et al. (2019) describes several ways that an individual’s sense of self could be altered if iBCIs come into common use, including changing the way they communicate with others, or altering their legal capacity, both of which may influence their ability to consent (Sample et al., 2019).

**Table 1:** Special risk and other ethical considerations for research involving iBCIs.

Risk category	Possible risks or ethical considerations	Brain-specific risks	Surgical risks: these include the standard risks associated with any surgical procedure, such as bleeding, infection, and anesthesia complications. Brain surgery carries additional risks due to the delicate nature of the brain, including potential damage to brain tissue, seizures, and stroke.	
	Device-specific risks: device migration can cause damage or require further surgery. The device could also malfunction, leading to inaccurate readings, loss of function, or unintended stimulation of other areas. Components of the implant can also break down within the brain, causing inflammation or other adverse reactions, or need to be surgical removed or replaced.
Neurological and psychological risks	Changes in personality or cognition: stimulation or interference with brain activity could lead to unintended changes in personality, mood, behavior, or cognitive abilities.
	Altered sense of agency or authenticity: participants may experience a diminished sense of control over their thoughts and actions or feel that their experiences are not authentic.
	Privacy and security concerns: neural data is highly sensitive and personal. There is a risk of unauthorized access to this data, which could be used to infer thoughts, emotions, or intentions.
Social and existential risks	Stigma and discrimination: participants may face social stigma or discrimination due to their implant.
	Identity issues: the use of a neural implant could raise questions about personal identity and what it means to be human.
	Dependence: participants may become dependent on the implant for daily functioning, leading to difficulties if the device malfunctions or is removed or deactivated.
Long-term uncertainties	Durability and maintenance: the long-term durability of neural implants is unknown. Participants may require additional surgeries or upgrades in the future.
	Effects of aging: the effects of aging, both of the device itself and the brain/human body, on the implant and surrounding brain tissue are uncertain and could lead to complications.
	Post-trial responsibilities: it is essential to address who is responsible for the ongoing care and maintenance of the implant after the research study ends, especially if the device is not yet commercially available. If the manufacturer closes or chooses to pursue a more commercially viable device, the participant may not have an option for ongoing care and maintenance of the implant after the research ends.

There are other risks unique to the use of iBCI that may be disclosed. For example, the long-term impacts of BCIs in general (Table 1) are not fully understood, adding an element of uncertainty to the research. Importantly, there are also concerns around what happens to an implanted device after the research study has concluded (Schönweitz et al., 2024). Currently, there is wide variability in what happens when an iBCI trial concludes, whether early or on time. If a participant has come to see the iBCI as a part of who they are, and has benefited from it, its absence likely will be felt as if a part of the participant is missing or lost. In this situation, some ethicists argue in favor of continued support (Goering et al., 2024; Lázaro-Muñoz et al., 2022). Participants may also move forward with a deactivated iBCI in their body, but there may be long term health implications, such as an inability to safely get an MRI or complications of device migration (Schönweitz et al., 2024). Explantation is another option for participants, but surgical explantation carries its own risks, similar to implantation (Dasgupta et al., 2024).

Beyond what information is included in a comprehensive informed consent process, researchers and regulatory authorities must also consider a participant’s ability to consent. Many individuals eligible to receive an iBCI may have a caregiver or guardian providing support with daily living activities, such as communication or mobility, or with medical decision making. For participants utilizing assisted or augmented communication support, it may be difficult to document consent, confirm participant comprehension, and support autonomous decision-making (Klein et al., 2018; Vansteensel et al., 2022). With this issue in mind, researchers can embed different mechanisms to check for understanding, such as asking the same question multiple times with slight variations, or focusing on yes/no questions to assess understanding (Vansteensel et al., 2022).

It is also important to consider the involvement of caregivers or legally authorized representatives (LAR) in the initial and ongoing consent process. The inclusion of a caregiver or LAR can be critical when working with an individual with a severe communication impairment. The presence of caregivers or LARs may facilitate a more favorable living situation for the research participant, providing practical support (Vansteensel et al., 2022). However, this involvement can also raise opportunities for potential for coercion by the caregiver, especially in situations where the caregiver is interpreting the participant’s communications and providing that to the study team. It raises the question of whether the participant is consenting because they personally want to be in the study, or if it is the caregiver who wants the participant to be involved.

Current federal regulations require that the prospective participant or their LAR be provided with sufficient study information and opportunity to consider participation, while also minimizing coercion or undue influence, before enrolling in a clinical trial. If consent is not obtained under these conditions, then it is not considered binding (21 CFR 50.20). The IRB is tasked with reviewing the consent process to ensure that the prospective participant is notified of the nature of the study and the investigational device, and that the consent is voluntary with consideration of any special variables that may be applicable to the specific research context. For example, if the proposed research will include adults lacking the capacity to consent, the IRB must determine if it is appropriate to include this population, taking into account relevant ethical principles for human subjects research (i.e., respect for persons, beneficence, and justice) (Forster and Borasky, 2018).

### Cyber security issues

While the primary cybersecurity risk in most research studies is loss of confidentiality, iBCI trials have the added risk of device hacking. Since iBCIs are real-time communication systems that connect the brain to an external device, they are susceptible to being hacked like any computer system. The bidirectional communication between an iBCI and the brain raises the possibility that actions may be performed that the individual did not intend. Importantly, this risk may not be immediately apparent to the IRB, since most IRBs lack cybersecurity experts on the panel and may not be aware of the additional risks with the use of an iBCI.

Cyberattacks on iBCIs present two specific concerns: obtaining the user’s private information without their consent and altering the user’s behavior by acting on neural activity. For example, current research indicates that it may be possible to extract specific Personal Identification Numbers (PIN) from EEG signals. It is also technologically feasible to design malicious applications which can record EEGs that can reveal correlations such as medical information or political action. This information could then be used in ways that could influence behavior such as direct interference (Brocal, 2023). Additionally, many iBCIs use wireless networks between the brain and the network. In this situation, it may be possible for a malicious attack to receive, read, and write signals unwanted by the user. Researchers have been able to experimentally simulate two types of neuronal cyberattacks that affect the biological activity of neurons. These cyberattacks are called Neuronal Flooding (FLO) and Neuronal Scanning (SCA). Among the main results of these experiments, it stands out that both types of cyberattacks are adequate to affect neural activity, with FLO being more effective in immediate terms and SCA in the long term. Another researcher was able to able to prevent neurons from producing spikes, in a type of cyberattack called Neuronal Jamming (JAM). If an iBCI is compromised, a cyberattack could inflict permanent brain damage or even cause death in the user (Bernal et al., 2022).

While cyberattacks on a wireless BCI are theoretical, the risk to the individual is significant but very difficult to quantify. As such, it becomes hard for an IRB to determine if the risk/benefit ratio for a study with an iBCI is appropriate. Being able to restore independence, through communication or the use of missing limbs will provide tangible and significant benefits to the users, but is that justifiable if the risk is that someone maliciously hacks into the system and causes permanent brain damage or is able to steal personal and intimate information (i.e., information is that related to ones thoughts, and beliefs, and therefore goes to the nature of self)?

Even if the study use of the device is being done in a secure facility, the risk of a cyberattack remains a concern since participants cannot simply remove the implant after study visits end. While current applications, and potential cyberattacks, of iBCI technology are relatively nascent, the threat of cyberattacks will increase as the technology advances and iBCI use becomes more widespread.

### Ensuring ethical iBCI research: IRB oversight

**Figure 2:**
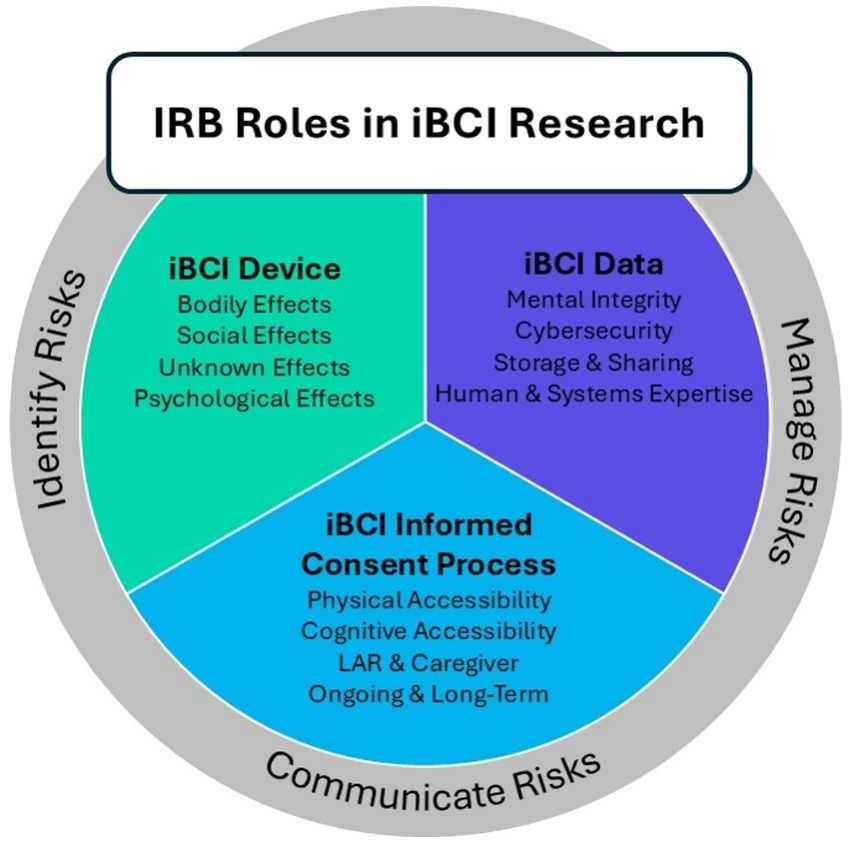
Illustration of framework for IRB review of iBCI human subjects research, illustrating how issues around informed consent, risk communication, and unique considerations around cybersecurity can be addressed by the IRB in a cohesive manner.

Having explored the complex ethical considerations surrounding iBCI research, the next step is to examine how these concerns translate into practical oversight. The IRB plays a critical role in ensuring the ethical and responsible conduct of iBCI research (Figure 2). When reviewing a research study involving an iBCI, the IRB is tasked with prioritizing participant safety and ethical treatment, adhering to the principles of respect for persons, beneficence, and justice as outlined in the Belmont Report. This includes a meticulous review of the informed consent process to ensure it satisfies the requirements set forth in 21 CFR part 50.

It is also important for the IRB to consider the unique study population while evaluating the consent process. Consent materials need to be accessible and understandable for all participants, regardless of communication or cognitive limitations. This may involve adapting materials for those with disabilities and setting realistic expectations about the capabilities of iBCIs. The IRB can also consider the existing health literacy of the target participant population, identifying where additional support may be necessary to ensure participants have enough of an understanding of the technology to make an informed decision about participation. This may include recommending the use of simplified participant-facing materials, visual aids, or other modifications to the consent process.

Enhanced regulatory frameworks could include tailored guidelines for iBCI submissions, specialized training for IRB members on ethical issues specific to neural technology, and a robust post-market surveillance system to monitor long-term neural outcomes. The FDA requires a 15-year follow-up period for gene transfer studies due to potential long-term risks such as cancer or genetic changes. Similarly, long-term follow up should be considered for iBCIs to evaluate risks, such as changes in neural pathways and possible alterations in personality. Researchers and regulatory authorities alike can also benefit from consulting with community advocacy groups, like the iBCI Collaborative Community (iBCI-CC), who specialize in identifying best practices in iBCI research and clinical care.

In accordance with Good Clinical Practice (GCP) guidelines and the Common Rule, researchers are obligated to ensure that potential participants are fully informed about the nature and scope of the study, including an understanding of the technology itself and possible changes to their behavior, emotional responses, or cognitive functions (U.S. Food and Drug Administration, 2014; ICH Harmonised Tripartite Guidelines, 2001). The information necessary to comprehend the unique risks associated with iBCIs, such as potential changes in personality, autonomy, and privacy, requires special attention. Clear and comprehensive risk communication, ongoing dialogue between researchers and participants, and a balanced presentation of the potential benefits and drawbacks of iBCI technology are crucial for maintaining the integrity of the informed consent process and upholding participants’ rights to make well-informed decisions about their involvement.

The potential psychological and cognitive changes associated with iBCIs underscore the importance of comprehensive and ongoing informed consent. Consent is not a one-time event but an ongoing process that must be revisited throughout the lifespan of the study. Current literature recommends that the informed consent process is best implemented by a team, including patient advocates and other non-clinicians. The research team should continuously engage participants to ensure they are adequately prepared for possible changes in their emotional and cognitive states (Klein et al., 2018).

Additionally, researchers must strive to minimize any undue influence that might sway a participant’s decision, such as overly optimistic portrayals of the technology’s capabilities or downplaying potential risks. Instead of merely managing therapeutic expectations, research teams should use them as an opportunity for ongoing dialogue with participants. This dialogue should center around the participant’s values and hopes, while ensuring a realistic understanding of the potential benefits of the research, particularly when the likelihood of success is low (Klein et al., 2018).

Uncertainties about the state of iBCI science, such as what is known and unknown, what participants are entitled to after a research study ends, and the long-term impact of implanted brain devices, need to be explicitly communicated as part of the ongoing consent process and revisited as necessary, especially if circumstances of participation change (e.g., iBCI devices evolve, companies exit the marketplace). As part of risk evaluation, the IRB should also confirm that robust cybersecurity measures are in place to prevent data breaches and unauthorized manipulation of brain activity. This aligns with current FDA guidance, which emphasizes the importance of threat modeling, cybersecurity risk assessment, and considerations with the use of third-party software component (U.S. Food and Drug Administration, 2023b).

## Conclusion

Neural implants, and iBCIs specifically, are a rapidly evolving area of research with the potential to offer therapeutic benefits to patients with a variety of conditions. While the promise of iBCI technology is significant, the unique nature of these devices demands careful ethical consideration and rigorous regulatory oversight to protect the rights and welfare of research participants. Ongoing ethical scrutiny and a dynamic regulatory framework are essential to ensure that iBCI research is conducted in a manner that prioritizes participant safety, respects individual autonomy, and promotes equitable access to the potential benefits of this transformative technology. This involves continuous re-evaluation of existing guidelines and the development of new, adaptive regulations that can keep pace with the rapid advancements in neural implant technology. By embracing a proactive and responsive approach to ethical oversight, the research community can harness the potential of iBCIs while safeguarding the well-being and dignity of those who participate, supporting equitable access to potential benefits, and helping advance this groundbreaking research. However, it is essential to acknowledge the significant hurdles IRBs must overcome to provide effective oversight in this rapidly evolving field. These include the complexities of evaluating potential risks to participants’ sense of self and autonomy, grappling with the long-term ethical implications of implanted devices, and ensuring robust cybersecurity measures are in place to protect sensitive neural data. Addressing these challenges will require ongoing dialogue and collaboration between researchers, ethicists, and regulatory bodies.
