# Supplementary material for: Mind the gap: bridging ethical considerations and regulatory oversight in implantable BCI human subjects research
Source: Front Hum Neurosci. 2025 Jul 23;19:1633627. doi: 10.3389/fnhum.2025.1633627 (PMC12325254; doi:10.3389/fnhum.2025.1633627)
Supplement: Supplementary file 1 [file Table_1.DOCX]

*Supplemental Table*

| Supplemental Table 1. Suggested framework for IRB review of iBCI human subjects research. | |
| --- | --- |
| Cybersecurity | Risk Assessment: The IRB will expect a thorough risk assessment that specifically addresses the potential for cyberattacks and data breaches. This should include:   - Identifying potential vulnerabilities in the iBCI system. - Describing the potential impact of a cyberattack on participants. - Detailing the measures taken to mitigate these risks (e.g., encryption, access controls, security testing).   Expertise: Given the technical complexity of cybersecurity, the IRB may seek external expertise to adequately assess the proposed security measures.  Data Management: The IRB will scrutinize data management procedures, including data storage, access, and sharing, to ensure participant privacy and data security. |
| Informed Consent | Accessibility: The IRB will ensure the informed consent process is accessible to all potential participants, including those with communication or cognitive impairments. This may involve:   - Ensuring participant materials use plain language and avoiding technical jargon. - Encourage the use of alternative formats (e.g., visual aids, audio recordings). - Support collaboration between members of the study team, patients, as well as caregivers or communication assistants when appropriate, in the consent process.   Realistic Expectations: The IRB will assess whether the informed consent process adequately addresses potential misconceptions about iBCI capabilities, ensuring participants have realistic expectations about potential benefits and limitations.  Ongoing Consent: The IRB may require researchers to implement an augmented ongoing consent process, allowing participants to revisit their decision to participate as the research progresses and their understanding of the technology evolves. |
| Risk Communication | Clarity and Completeness: The IRB will ensure that the informed consent document and other participant materials clearly and comprehensively explain the unique risks associated with iBCIs, including:   - Potential changes in personality, cognition, or sense of self. - Risks to privacy and autonomy. - Long-term uncertainties and potential need for future surgeries or device upgrades.   Contextualization: The IRB will expect researchers to present these risks in a balanced way, weighing them against the potential benefits of the iBCI and alternative treatment options.  Ongoing Communication: The IRB may encourage open communication between researchers and participants throughout the study to address any emerging concerns or questions about risks and benefits. |
